# Validating the Operational Bias and Hypothesis of Universal Exponent in Landslide Frequency-Area Distribution

**DOI:** 10.1371/journal.pone.0098125

**Published:** 2014-05-22

**Authors:** Jr-Chuan Huang, Tsung-Yu Lee, Tse-Yang Teng, Yi-Chin Chen, Cho-Ying Huang, Cheing-Tung Lee

**Affiliations:** 1 Department of Geography, National Taiwan University, Taipei, Taiwan; 2 Taiwan Typhoon and Flood Research Institute, Taipei, Taiwan; Centro de Investigacion Cientifica y Educacion Superior de Ensenada, Mexico

## Abstract

The exponent decay in landslide frequency-area distribution is widely used for assessing the consequences of landslides and with some studies arguing that the slope of the exponent decay is universal and independent of mechanisms and environmental settings. However, the documented exponent slopes are diverse and hence data processing is hypothesized for this inconsistency. An elaborated statistical experiment and two actual landslide inventories were used here to demonstrate the influences of the data processing on the determination of the exponent. Seven categories with different landslide numbers were generated from the predefined inverse-gamma distribution and then analyzed by three data processing procedures (logarithmic binning, LB, normalized logarithmic binning, NLB and cumulative distribution function, CDF). Five different bin widths were also considered while applying LB and NLB. Following that, the maximum likelihood estimation was used to estimate the exponent slopes. The results showed that the exponents estimated by CDF were unbiased while LB and NLB performed poorly. Two binning-based methods led to considerable biases that increased with the increase of landslide number and bin width. The standard deviations of the estimated exponents were dependent not just on the landslide number but also on binning method and bin width. Both extremely few and plentiful landslide numbers reduced the confidence of the estimated exponents, which could be attributed to limited landslide numbers and considerable operational bias, respectively. The diverse documented exponents in literature should therefore be adjusted accordingly. Our study strongly suggests that the considerable bias due to data processing and the data quality should be constrained in order to advance the understanding of landslide processes.

## Introduction

Landslides are an inevitable phenomenon in mountainous regions [1]–[3] and they are one of the most widespread and underrated natural hazards on earth [4]. They generate abundant sediments and profoundly affect the sediment transport, downstream ecosystem and topographic evolution [3]–[5]. For quantitatively assessing the landslides impacts on a regional scale, landslide inventories are the foundation for regional hazard assessment, and estimations for erosion rates, sediment volume, sediment fluxes, and the physics of landsliding [6]–[9]. It is well recognized that the distribution of landslides in a region is characterized by much fewer large landslides and plentiful of small ones and its frequency-area distribution follows the power law relation in most cases [10] [3] [11]. Thus the distributions which can describe the exponent decay have been widely used for landslide studies. For example, the cumulative frequency-area distribution, double-Pareto distribution, and inverse gamma distribution have all been proposed for estimating landslide area [10]–[12]. Those distributions have their own characteristics and parameters while the slopes of the exponent decay are comparable theoretically. Thus comparing the exponent slopes among different landslide inventories is a way to advance understanding of landslide processes (e.g. to characterize the regional features and the triggering magnitudes).

In power-law-like distributions, the exponent slope, ρ, dominates the inferences, particularly for the large landslides and the total landslide area. Although there are several approaches being used to estimate the exponents in other fields, landslide related literature rarely discussed how the choice of approaches (e.g. binning method and bin number) and how the quantity of landslide number influence the exponent estimation. Meanwhile, previous studies argued that the exponent slope should be universal and irrelative to mechanisms [10]. Guzzetti's studies suggest that the estimated exponent is around 1.4–1.5 and is independent to regions and mechanisms [1] [8] [12], while the estimated exponents in other studies are quite diverse [3] [13]–[16]. Therefore, it becomes important to ascertain the hypothesis of the “universal exponent”.

In this study, an elaborate statistical experiment and two actual landslide inventories were used to investigate the operational bias induced by data processing and to validate the hypothesis of the universal exponent. Seven categories with different landslide numbers (defined as the sum of individual landslide scars in a region) were generated from the predefined inverse-gamma distribution and then analyzed by three data processing procedures (logarithmic binning, LB, normalized logarithmic binning, NLB and cumulative distribution function, CDF). While applying LB and NLB, five different bin widths were also considered. Then the maximum likelihood estimation was used to estimate the exponents. Besides, the documented exponents in previous literature and two realistic landslide inventories were used for testing the hypothesis of the “universal exponent”. Our results may help to advance the understanding of the landslide frequency-area distribution, to provide the common ground for the inter-comparison of the estimated exponents, and to constrain the uncertainty for further applications, such as landslide volume estimation, sediment flux calculation, and landslide risk assessment.

## Background and Methods

Landslide inventory maps derived from aerial photos or field surveys can record the discernible features of the location, date of occurrence, and other geomorphic features; yet for many reasons (e.g. the scale of base maps and photos, operational standard of identification, and the resources of field confirmation and survey) the landslide inventory maps are incomplete and inevitably inconsistent [12] [17] [6]. Despite of the technical uncertainty, binning the observed data as a histogram to characterize the landslide frequency-area distribution and the exponent decay, is an intuitive way to quantify the landslide frequency. In fact, the arbitrary binning methods may make the accuracy and precision of the estimated exponent doubtful especially when the data spans several orders. In a landslide frequency histogram, the landslide area varying from 10^−5^–10^1^ km^2^ with large landslide occupying less than 1.0%, even to 0.1% is common. Besides, two notifications should be addressed as analyzing the landslide frequency-area distribution. First, the exponent slope is a negative value; however, the negative sign is presumably assigned in the distribution formulae and thus positive exponent values are commonly documented. Second, both of the non-cumulative and cumulative landslide frequency-area distributions follow exponent decay, but the cumulative distribution, the integral of probability distribution is in fact (ρ+1), not ρ. The values of the documented exponents and the basic information of cases in literature were collected in Table 1. Note that the ρ values are varied and do not support the hypothesis of the “universal exponent”.

**Table 1:** Estimated exponents and backgrounds of the landslide inventories from literature reviews.

Case ID	Country	Location	Event/Historical	Landslide number	Bin number^1^	Estimated exponent^2^	Reference
A	USA	California	Earthquake	11,111	∼37	1.40^a^	Malamud et al. [12]
B	Italy	Umbria	Snowmelt	4,233	∼32	1.40^a^	Malamud et al. [12]
C	Guatemala	Motagua	Rainstorm	9,594	∼45	1.40^a^	Malamud et al. [12]
D	Taiwan	Ma-An	Historical	1,040	unknown	1.40^a^	Malamud et al. [12]
E	Bolivia	Challana	Historical	1,130	unknown	1.40^a^	Malamud et al. [12]
F	New Zealand	WSA	Historical	5,086	∼40	1.44–1.48^b^	Stark and Hovius [11]
G	Taiwan	Central Range	Historical	1,086	∼17	1.11^b^	Stark and Hovius [11]
I	USA	Walker Creek	Event and Historical	416, 688	∼17, ∼15	1.12∼–1.15^a^, 1.22∼–1.29^a^	Florsheim and Nichols [28]
J	UK	Loughborough Inlet	Event	101	unknown	1.51∼1.77^b^	Guthrie and Evans [29] [30]
K	Taiwan	Shihmen	Historical	unknown	23–30	1.86–2.94^a^	Tsai et al. [15]
L	Italy	Umbria	Historical	16,809	unknown	1.50^c^	Guzzetti et al. [1]
M	Italy	Lombardy	Historical	2,390	45	1.77^a^ or 1.50^b^	Guzzetti et al. [8]
7	UK	Queen Charlotte	Historical	475, 140	∼15, ∼15	0.80^c^, 2.20^c^	Martin et al. [14]
O	New Zealand	Southwest	Historical	778	∼27	1.44^c^	Korup [6]
P	Morocco	Rif mountains	Historical	759	∼14	1.57^c^	Rouai and Jaaidi [31]
Q	Japan	Alkishi Ranges	Historical	3,424	CDF	1.0^c^	Pelletier et al. [10]
R	USA	Califoria	Earthquake	11,000	CDF	0.6^c^	Pelletier et al. [10]
X	Guatemala	Motagua	Rainstorm	9,594	25∼117	1.41^a^	This study
Y	Taiwan	Gaoping	Rainstorm	3,841	25∼117	1.12^a^	This study

1. Bin number is counted and estimated from plots in the individual papers.

2. Estimated exponent: the superscript a, b, and c indicated the estimated method which is derived from inverse gamma, double pareto, and power law, respectively.

### Experiment Design for Exponent Estimation

A total of seven categories with different landslide numbers, 100, 300, 600, 1000, 3000, 6000 and 10,000, were designed to assess the operational bias. The range of landslide number was chosen in accordance with the cases in literatures (Table 1). For each category, 1,000 landslide inventories were generated from inverse gamma distribution with the fixed parameters (ρ = 1.40, a = 1.28×10^−3^ km^2^ and s = −1.32×10^−4^ km^2^, see the next section). This parameter set was suggested by Malamud et al. [12] who stated that this set is applicable and universal for most of the landslide inventories. These generated inventories were then applied onto different data processing procedures for estimating the exponent.

In general, the common way to represent the landslide frequency-area distribution is to construct a histogram from inventories. To construct a histogram, the interval covered by the data is separated by several subintervals as “bins”. However, there is no simple and proper rule to decide the bin width apart from trial-and-error or arbitrary determination. Linear binning methods are widely used. This method is to decide a constant width for the bins and then to count the observation fallen within the bins to construct the histogram while this is improper for observations spanning several orders. Consequently, two other modified binning methods were proposed. The first one is the logarithmic binning (LB) which is similar to the linear binning but is operated on a logarithmic scale where the bin width is held constant, 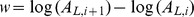. This method thus reduces the numerous zeros in linear binning and combines low-count bins at larger landslide area. However, its disadvantage is that the observation numbers within each bin is affected by the changing actual width. This problem can be dealt with normalizing the observation numbers within each bin by the bin width, and it is called normalized logarithmic binning (NLB). NLB converts the observation numbers to densities, i.e., observation number per area unit. In practice, NLB commonly selects a fixed multiple, *b*, and calculates the bin width as 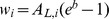 [18] [19]. In addition to the mentioned binning-based methods, an alternative way to estimate the exponent is using the cumulative distribution function (CDF) which ranks the whole dataset to calculate the observed cumulative distribution. Since this method uses the whole dataset without any transformation, no information within inventory would be generalized or lost. After data processing by the three methods, the maximum likelihood estimation (MLE) is further applied to estimate the exponents. This is one of the preferred approaches among researchers (e.g. [19]–[20]) for estimating the exponents. MLE determines the unknown parameters which can maximize the likelihood of the given distribution. The performance measure, likelihood, is to summarize the product of the probabilities of each observation. The more approximate the estimated parameters are to observations, the higher likelihood is obtained.

For logarithmic binning (LB), given the landslide area from −4.0∼1.0 on the logarithmic scale, five intervals, 0.05, 0.125, 0.25, 0.375, and 0.50 were set as bin width, resulting in 101, 41, 21, 14, and 11 bins, respectively. For normalized logarithmic binning (NLB), five multiples, 0.1, 0.2, 0.3, 0.4, 0.5 were set and thus the numbers of the bins were 117, 59, 40, 30, and 25, respectively. LB, NLB and CDF, totally three data processing procedures were then applied onto the 7 categories to assess the operational bias in the estimated exponents.

### Inverse Gamma Distribution

As mentioned in the introduction, many power-law-like distributions have been proposed for landslide frequency-area distribution. In this study, the three-parameter inverse-gamma distribution [12] [17] was applied as shown below:
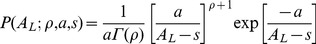
(1)where A_L_ [km^2^] means landslide area and ρ primarily controls the power-law decay for medium and large landslides. The parameter *a* [km^2^] controls the location of the maximum probability and *s* [km^2^] controls the shape of the probability for small landslides. This parameter shifts the probability for small landslides while it becomes trivial with the increase of landslide area because the small *s* value cannot alter the probability for large landslides. Therefore, it can be used to detect the characteristics of small landslides. The parameter *a* dominates the maximum probability, the so called “rollover” which may be relevant to the detection limitation [11] or the minimum requirement of the accumulated drainage area of the first order stream [1]. Besides the two parameters for small landslides, the parameter ρ primarily controls the large landslides and hence the total landslide area. This parameter coincides with the exponent in “sandpile” and “self-organized cellular-automata” model [10] [13]. However, the gap between the statistical models and the governing physics in landsliding is undetermined and needed to be explored further [1].

### Two Landslide Cases

**Figure 1:**
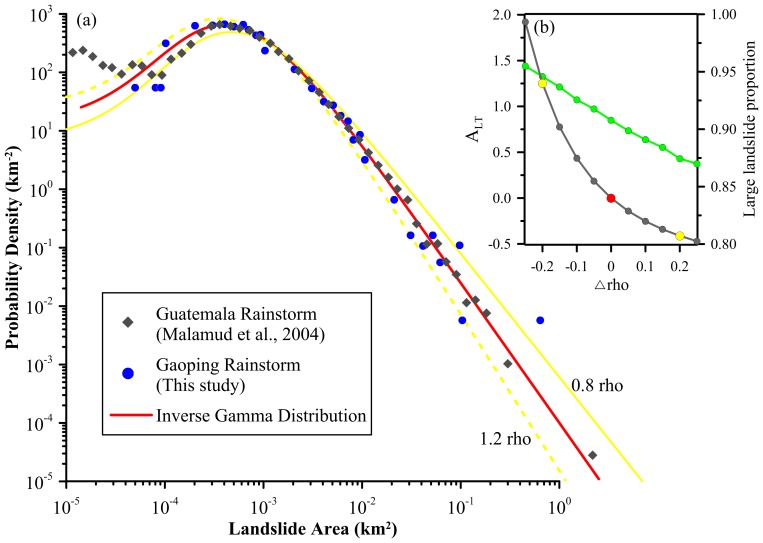
Inverse gamma distribution for landslide frequency-area estimations. (a) The gray diamond and blue circle indicate the landslide inventories of the Guatemala and Taiwan case. The red line is the reference distribution with ρ = 1.4, *a* = 1.28×10^−3^ km^2^, *s* = 1.32×10^−4^ km^2^. The solid and dashed yellow lines represent the distribution with 0.8ρ and 1.2ρ, respectively. Their corresponding *r* ^2^ to the reference reach 0.973 and 0.976, respectively. (b) Change of total area and large landslide proportion is associated with the change of the exponent.

Two catastrophic cases were used to demonstrate the operational bias induced by data processing (Figure 1). One landslide inventory was triggered by Hurricane Mitch [21] in Motagua, Guatemala and the other one induced by Typhoon Morakot [22] occurred in Gaoping River watershed, Taiwan. The details of the two landslide inventories can be referred to the cited literature. Since the parameter ρ is a dominant parameter and the other two parameters only show the significant change in small landslides, only the sensitivity of parameter ρ was illustrated in Figure 1b. This figure shows that the inverse gamma distribution could describe the frequency-area distributions of landslide inventories quite well with *r* ^2^>0.95. Besides, with the increase of ρ, the probability of small landslides would significantly increase and large ones decrease. The sensitivity result shows that with 20% change in ρ, the fitted curve is not changed very distinctly. In fact, the distribution of 0.8 and 1.2ρ are much similar to the reference with the *r* ^2^ values over 0.97. However, the responses of total landslide area for 0.8 and 1.2ρ are factors of 2.25 and 0.59, compared to the reference. The large landslide proportions from 0.8 to 1.2ρ decreased from 0.95 to 0.87. It reveals that even when the changes of ρ were subtle in *r* ^2^ the changes for the inferences (e.g. total landslide area and large landslide proportion) are substantial. Furthermore, the landslide volume estimation is exponentially proportional (the exponent is around 1.0–1.5) to landslide area, indicating the change is amplified substantially. The sensitivity result also indicates that the increase of ρ values results in the reduction of large landslide probability, and thus infers less large landslides and smaller total area. It also highlights the necessity of accurate and precise estimations for the exponent slope.

## Results and Discussions

**Figure 2:**
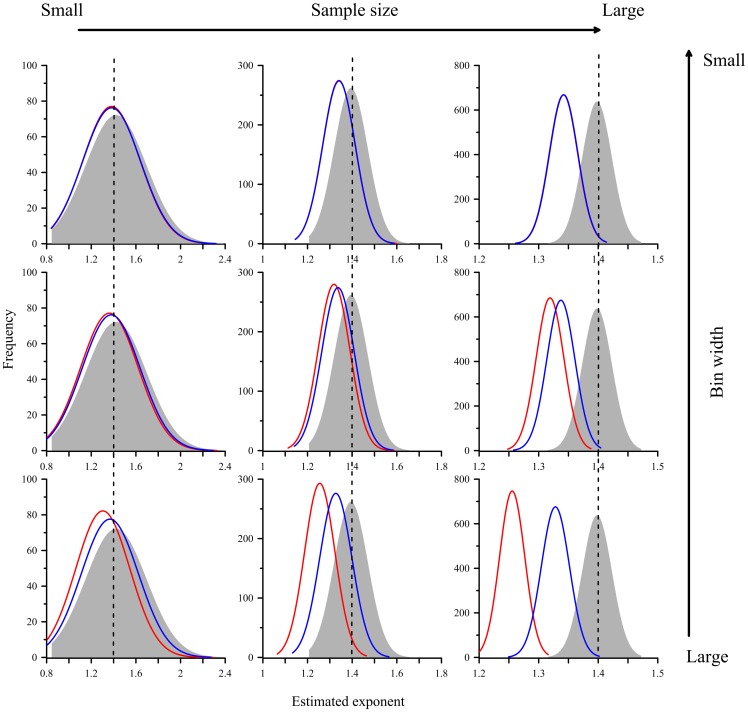
Effect of sample size and bin width on the exponent estimation. The red and blue distributions are derived from LB and NLB method, respectively, and the gray zone is derived from CDF. The dashed line represents parameter ρ = 1.4.

**Figure 3:**
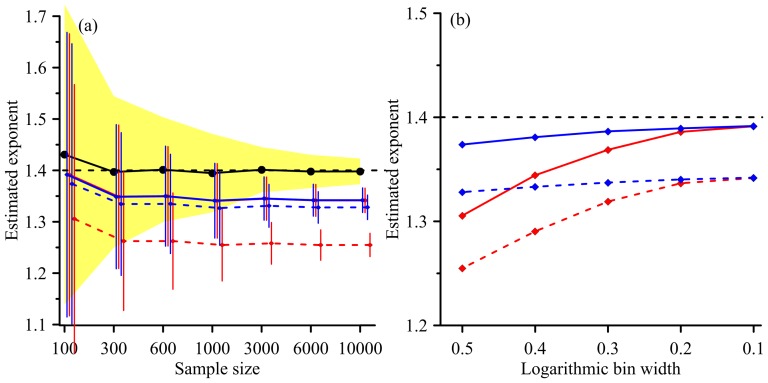
Effect of sample size and bin width on the estimated exponent. (a) The black lines and yellow zone are the mean standard deviation of estimated exponents derived from CDF, separately. The solid and dashed red curves are the results of LB with bin number = 101 and 11, respectively. The solid and dash blue curves are the results of NLB with bin number = 117 and 25, respectively. The vertical lines represent the standard deviations at the given sample size. (b) The black dashed line represents the reference. The solid red and blue curves indicate the results of LB and NLB, respectively, with landslide number = 10000. The dashed red and blue curves indicate the results of LB and NLB, respectively, with landslide number = 100. The standard deviations are not shown on (b)

Making accurate and precise inference from samples (e.g. landslide inventories) to population (e.g. earth systems) is the ultimate concern of a statistical approach. In landslide issues, the sample size (landslide number), binning methods, and bin numbers play primary roles in histogram making and exponent estimating. The estimated exponents derived from different combinations of sample size, binning methods and bin numbers are illustrated in Figure 2 and Figure 3. There are several general rules that are worth stating here. Firstly, sample size dominates the standard deviation of the exponent which is independent of the binning methods and binning numbers. It means that landslide inventories containing only a few landslides are pretty uncertain to infer a precise exponent of population. Even if the landslide frequency-area distribution in earth system is universal, but unknown, the exponent obtained from limited available landslide inventories, particularly those with sparse landslides, is still skeptical. Secondly, the LB performs poorly and the exponents estimated by it are significantly biased. The bias increases significantly with the increase of bin width and landslide number. NLB performs much better than LB, particularly distinct for wide bin width and inventories with abundant landslides. Improvement through binning-number adjustment can be expected. However, this indicates that binning-based methods are sensitive to a variety of arbitrary decisions (e.g. bin width), and may not be easy to justify the estimated exponents. Generally, binning-based methods which may lose some information when binning the original distribution is expected to perform poorly [23] [24]. Thirdly, CDF outperforms in terms of the accuracy of estimated exponents [25]. CDF which utilizes all information (no generalization) of the original data is capable of presenting satisfactory performances, though it may not provide visual comparisons.

**Figure 4:**
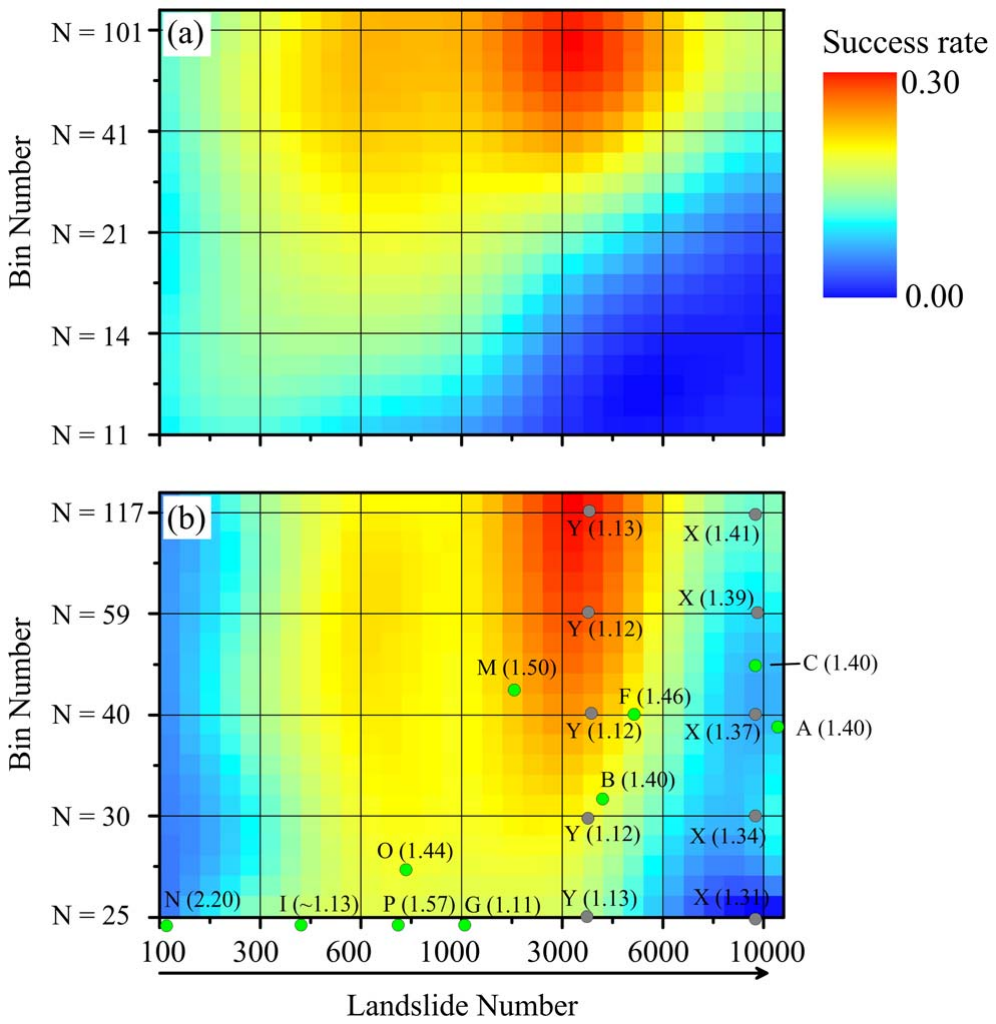
Success rate of (a) LB- and (b) NLB-estimated exponent associated with the landslide number (sample size) and bin width. The success rate is defined as the counts of exponents which are estimates fallen successfully within 1.4±2.5% over the 1000 instances. The dots are the exponent values retrieved from literature (see table 1 and text).

We further introduced an indicator, success rate, into our analysis. The success rate is defined as the count which estimates the exponent successfully (within the range of 1.4±2.5%) over the 1000 landslide inventories. Briefly speaking, this indicator simultaneously deliberates accuracy and precision. The success rates of LB and NLB are shown in Figure 4. We find that there is a hotspot of success rate (approximately 0.30) positioned in landslide number between 1000–6000 with bin number around 50–100 while the NLB is a more applicable method than LB. The success rate of CDF which is not shown because it represents a plane (the method is independence of bin numbers) gradually increases from 0.114 to 0.831 with the increase of landslide number. We also find two areas of low success rates on the left and right side which are due to insufficient landslide numbers and the underestimation of operational bias, respectively. It implies that even if we can accurately estimate the exponent for a sample (landslide inventory with smaller landslide number), we still cannot ascertain the precision of population inference. On the other hand, the precision of the estimated exponent is acceptable for inventories with large number of landslides, but the operational bias damps the accuracy. It implies that the accumulation of landslide inventories and the improvement of the operational bias are necessary as making the inference from landslide frequency-area distribution.

The estimated exponents of the Guatemala and Taiwan case from different NLB treatments are illustrated in Figure 4b as well. It shows that the operational bias of the Guatemala case is distinct in which the exponents change from 1.41 to 1.31 resulting in 27.9% bias in the total landslide area estimation (see our preceding sensitivity analysis). By contrast, the estimated exponents in the Taiwan case remain stable due to the case fallen in the hotspot coincidentally. However, the values (∼1.12–1.13) differ far from the universal exponent (1.40–1.50).

**Figure 5:**
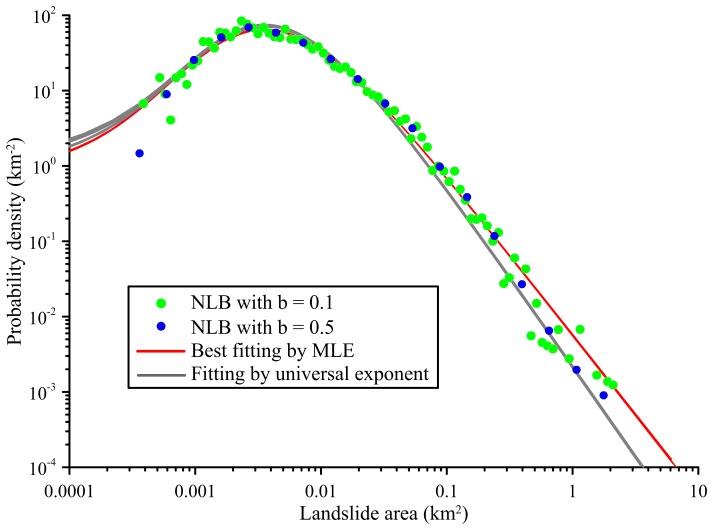
Probability density functions of Gaoping, Taiwan landslide inventory. The green and blue dots are produced by NLB with b = 0.1 and 0.5, respectively. The red lines are fitted by MLE and the gray lines are derived from the universal exponent (ρ = 1.4) and the corresponded *r* ^2^ values for b = 0.1 are 0.949 and 0.948, respectively.

All the documented cases, including the estimated exponents, bin numbers, and landslide numbers shown in Table 1, are put on figure 4b to discuss the universal exponents. As the table shows, the landslide number ranges from 101∼11,111 and the bin number retrieved from literature is around 14∼45. Note that the bin number was visually counted from plots in literature. It may not be the same number that the literature used. Nevertheless, it reveals that the documented exponents may be derived from insufficient bin number which results in the considerable operational bias. Besides, most of the exponents are estimated from 1.10 to 1.57, except for #N case (ρ = 2.20) which is used for gully erosion, not for landslide [14]. If this value is true, it may imply that the area variation of gully erosion is much larger than that of landslides. Another contrasting case is rock fall which has a low exponent indicating the area variation in rock fall is less than that in landslide [26] [27]. Regardless of the gully erosion and rockfall cases, it seems that there are two groups of exponent values for landslides. One is 1.40–1.50 and the other is ∼1.10. For example, Stark and Hovius [11] estimated the exponent in Ma-An and Wan-Li watershed in Taiwan to be around 1.10 while a large exponent of 1.40 was reported in Malamud et al. [12]. Therefore, the Gaoping, Taiwan case was selected for discussing the contradiction (Figure 5). In this case, the results of different binning width (e.g. b = 0.1 and 0.5) are comparable and the exponents derived from MLE estimation are ∼1.10 with the *r* ^2^ = 0.949, consistent with Stark and Hovius' work. However the result from the universal exponent (ρ = 1.40) is visually satisfactory with *r* ^2^ = 0.948. Although the estimated exponent derived from MLE is unbiased and has the highest likelihood, the incompleteness of actual landslide inventory may result in considerable bias as well. Therefore, the universal exponent cannot be rejected with regard to data quality. In this regard, the hypothesis of universal exponent is still valid and the key point should turn to establish a common platform for evaluating the exponents. Such a platform is valuable in discussing whether the exponents are universal in different regions or they are controlled by the physiographic settings [1] and it is beneficial in constraining the operational bias for further landslide volume estimation as this study presented.

## Summary

Landslide frequency-area distribution is widely used to infer the total landslide area (volume) for sedimentation issues in landslide-dominant region. We revealed that the exponent estimation in landslide frequency-area distribution strongly depends on binning methods, bin numbers and landslide number. It means that the estimated exponents in the previous studies should be reflected again. The uncertainties of the documented exponents which is influenced substantially by even a subtle change in ρ should be constrained quantitatively before further applications (e.g. landslide area and volume). Our results show that CDF-estimated exponent outperforms the two binning-based methods. The NLB method is recommended because it is less operational bias, if binning is necessary. Two groups of exponents (1.10 and 1.40) found from previous literature seem to draw a shadow of a doubt on the hypothesis of the universal exponent. The discrepancy may result from the operational bias in data processing or unmeasurable data quality. Consistent exponent estimation derived from the same data processing is therefore suggested to form the basis for comparing the frequency-area distribution in order to advance the understanding of landsliding physics. Meanwhile, an uncertainty analysis targeting on giving thought to data quality, reducing the influence of incompleteness in landslide inventory and providing a complimentary confident interval should be explored further.
